# How Are Self-Reported Physical and Mental Health Conditions Related to Vaping Activities among Smokers and Quitters: Findings from the ITC Four Country Smoking and Vaping Wave 1 Survey

**DOI:** 10.3390/ijerph16081412

**Published:** 2019-04-19

**Authors:** Lin Li, Ron Borland, Richard J. O’Connor, Geoffrey T. Fong, Ann McNeill, Pete Driezen, K. Michael Cummings

**Affiliations:** 1 Nigel Gray Fellowship, Cancer Council Victoria, Melbourne, Victoria 3004, Australia; Ron.Borland@cancervic.org.au; 2 Department of Health Behavior, Roswell Park Comprehensive Cancer Center, Buffalo, NY 14203, USA; Richard.O’Connor@roswellpark.org; 3 Department of Psychology, University of Waterloo, Waterloo, ON N2L 3G1, Canada; geoffrey.fong@uwaterloo.ca (G.T.F.); prdriezen@uwaterloo.ca (P.D.); 4 School of Public Health and Health Systems, University of Waterloo, Waterloo, ON N2L 3G1, Canada; 5 Ontario Institute for Cancer Research, Toronto, ON M5G 0A3, Canada; 6 National Addiction Centre, King’s College London, London WC2R 2LS, UK; ann.mcneill@kcl.ac.uk; 7 Department of Psychiatry and Behavioral Sciences, Medical University of South Carolina, Charleston, SC 29425, USA; cummingk@musc.edu

**Keywords:** risk of tobacco use, health conditions, smoking cessation, vaping, survey research

## Abstract

This study examines whether having health conditions or concerns related to smoking is associated with use of vaping products. Data came from the 2016 wave of the International Tobacco Control Four Country Smoking and Vaping Survey. Smokers and recent quitters (*n* = 11,344) were asked whether they had a medical diagnosis for nine health conditions (i.e., depression, anxiety, alcohol problems, severe obesity, chronic pain, diabetes, heart disease, cancer, and chronic lung disease) and concerns about past and future health effects of smoking, and their vaping activities. Respondents with depression and alcohol problems were more likely to be current vapers both daily (Adjusted odds ratio, AOR = 1.42, 95% confidence interval, CI 1.09–1.85, *p* < 0.05 for depression; and AOR = 1.52, 95% CI 1.02–2.27, *p* < 0.05 for alcohol) and monthly (AOR = 1.32, 95% CI 1.11–1.57 for depression, *p* < 0.01; and AOR = 1.43, 95% CI 1.06–1.90, *p* < 0.05 for alcohol). Vaping was more likely at monthly level for those with severe obesity (AOR = 1.77, 95% CI 1.29–2.43, *p* < 0.001), cancer (AOR = 5.19, 95% CI 2.20–12.24, *p* < 0.001), and concerns about future effects of smoking (AOR = 1.83, 95% CI 1.47–2.28, *p* < 0.001). Positive associations were also found between chronic pain and concerns about past health effects of smoking and daily vaping. Only having heart disease was, in this case negatively, associated with use of vaping products on their last quit attempt (AOR = 0.72, 95% CI 0.43–0.91, *p* < 0.05). Self-reported health condition or reduced health associated with smoking is not systematically leading to increased vaping or increased likelihood of using vaping as a quitting strategy.

## 1. Introduction

Cigarette smoking harms almost every organ of the human body and causes a broad range of diseases, including cardiovascular disease, respiratory disease, cancers, some mental health issues, and other health problems [1,2]. Having adverse health effects linked to smoking should motivate action to quit, and it may also motivate seeking out potentially less harmful alternatives, including use of nicotine vaping products (NVPs), sometimes called electronic cigarettes. NVPs deliver nicotine without the vast bulk of the other toxicants [3,4]. Nicotine in the doses people voluntarily self-administer has fewer direct harms, but if delivered in ways that result in rapid uptake to the brain, can be dependence-forming [4,5]. Countries in this study have different approaches to regulating NVPs. Overall, the United States and England have fewer restrictions on the marketing of NVPs compared to Australia and Canada. The aim of this paper is to find out if having or fearing smoking-related harms is associated with use of NVPs in these four countries.

Having smoking-related illnesses such as cancers has been found to be associated with high rates of smoking cessation [6,7]. Persons diagnosed with hypertension and chronic heart disease, chronic obstructive pulmonary disease (COPD), and diabetes have also been found to have a greater desire to quit smoking, although this has not always translated into higher rates of smoking abstinence [7,8,9,10,11,12,13]. 

People with mental health problems (e.g., depression, anxiety) are more likely to smoke, and to smoke more heavily, than the general population [2,14,15]. The smoking prevalence among people with clinical depression is approximately twice as high as that among the general population [16,17]. Comorbid disorders are associated with higher proportions of heavy smoking [2]. Smoking rates have come down in the general population over the last few decades but not among people with indicators of longstanding mental disorders or recent psychoactive medication usage [18]. Results from general population surveys also indicate that smokers have higher rates of depression than nonsmokers—that is, smoking may increase risk of depression [2,19,20]. However, more complex analyses suggest that there is not a causal relationship, findings being more consistent with a self-medication hypothesis, whereby smoking is used to alleviate symptoms of depression [21]. Severe depression seems to make it difficult for smokers to quit even though they are aware of the long-term harms of smoking [2], although they may be more likely to make quit attempts [22]. Cessation rates remain consistently lower for smokers diagnosed with depression than for smokers in the general population [23]. They may also be more interested in using alternative nicotine products, such as NVPs. 

We hypothesized that those having health conditions or concerns about them related to smoking would be generally more likely to use/keep using NVPs to avoid the adverse effects of smoking and would thus be more likely to use them as a smoking cessation strategy.

## 2. Materials and Methods 

### 2.1. Data Source and Participants

The data came from Wave 1 of the International Tobacco Control (ITC) Four Country Smoking and Vaping (4CV) Survey conducted in Australia (AU), Canada (CA), England (EN), and the United States (US) from July to November 2016. The valid core sample (*n* = 12,022) consisted of the following respondent groups: (1) Adult smokers and quitters who responded to previous ITC-4C surveys [24] and were successfully re-contacted online; (2) current smokers and past smokers who had quit smoking in the past 2 years and were newly recruited in 2016 through online probability-based consumer panels; and (3) newly recruited current NVP users (i.e., vapers). The weighting was designed to make the sample representative of smoking, recent quitting, and vaping product users in each country [22]. Because the current study is mainly concerned about the associations between a range of smoking-related health problems and vaping among cigarette smokers and recent quitters who stopped smoking in the past 2 years, a total of 72 cases of nonsmokers (never smokers) and 606 cases who were re-contacted from previous ITC-4C survey (out of the first group mentioned above) but who had been quit for more than 2 years (long-term quitters) were excluded from analysis. Hence, our analytic sample consisted of a total of 11,344 respondents (Australia: *n* = 1490; Canada: *n* = 3576; England: *n* = 4220; and the US: *n* = 2058). Table 1 presents sample characteristics by vaping status. A more detailed description of sampling methods and original sample size for each country can be found elsewhere [24,25,26]. 

### 2.2. Measures

#### 2.2.1. Self-Reported Health Problems 

Respondents (both current smokers and recent quitters) were asked whether they were currently being treated for, or had been diagnosed (current diagnosis) with health problems of three types that are related to smoking cigarettes: (1) Those that are mental health related (i.e., depression, anxiety, alcohol problems); (2) those where there are strong causal links (heart disease, cancer (excluding non-melanoma skin cancer) and chronic lung disease (e.g., chronic bronchitis and emphysema)); and (3) those with more complex associations (severe obesity, chronic pain, and diabetes). Their answers were coded as “yes, selected” vs. “not selected/don’t know”. In England, respondents were asked about lung cancer and other types of cancer separately; and instead of chronic lung disease, asked about four specific conditions: Asthma, emphysema, chronic bronchitis or tuberculosis. Reporting any (either) was coded as having the general condition. We also conducted supplementary analysis on a composite of having any of the three mental health conditions (depression, anxiety, and alcohol problems) and any of the three conditions most commonly caused by smoking (cancer, heart disease, and lung disease). 

In addition, participants were asked “To what extent, if at all, has smoking cigarettes damaged your heath?”, and (current smokers only) “How worried are you, if at all, that smoking cigarettes will damage your health in the future?” The response options for both were: “not at all”, “just a little”, “a fair amount/moderately”, “a great deal”, and “don’t know”. Responses were recoded into at least moderately vs. all lesser responses and “don’t know”. 

#### 2.2.2. Vaping and Quitting Related Measures

All respondents were asked whether they had ever used a vaping product, and those ever used were asked whether they currently vaped (“daily/weekly/monthly” vs. not current: “less often than monthly/not at all/don’t know”). Current vapers were then asked whether they planned to continue vaping or to stop using sometime in the foreseeable future (“definitely/probably keep using” vs. “definitely/probably stop using/might or might not keep using/don’t know”). 

Quit attempts were assessed by asking current smokers who reported ever making quit attempts: “How many times, if any, have you tried to quit in the past 12 months?” (“no attempt” (including never tried) vs. “1 or more attempts”). Those who reported having made at least one quit attempt in the last 12 months were then asked about their use of vaping products (“yes” vs. “no/don’t know”) for their last quit attempt. 

#### 2.2.3. Other Covariates

Demographic measures were sex (male, female) and age (18–24, 25–39, 40–54, 55 and older; but in analyses of interactions, it was recoded as “<40 years” vs. “40 year or older”). Due to the differences in economic development and educational systems across countries, only relative levels of income and education were used. “Low” level of education referred to those who completed high school or less in Canada, the US, and Australia, or secondary/vocational or less in England; “moderate” meant community college/trade/technical school/some university (no degree) in Canada and the US, college/university (no degree) in England, or technical/trade/some university (no degree) in Australia; and “high” referred to those who completed university or postgraduate studies in all countries. Household income was also grouped into “low” (less than $30,000 (country-specific dollars) (£30,000 in England) per year), “moderate” ($30,000 to $59,999 (£30,000 to £44,999 in England)), “high” categories (equal to or greater than $60,000 (£45,000 in England)), and “no information”. Cigarette smoking status was also asked and recoded into current smokers (at least monthly) and recent quitters (all others). 

### 2.3. Data Analysis

The analysis was weighted, and the stratified sampling design was accounted for. The prevalence/percentages of key measures are reported. To compare self-reported health conditions by demographics and vaping activities, and to examine the associations between health conditions and vaping, for each health condition separately, we employed logistic regression, controlling for covariates which were age, sex, education, income, smoking status, and country. We also tested for interactions by health condition with smoking status, sex, age, and country but only included interaction effects in the models we report when they were significant. In all analyses, a *p*-value < 0.05 was considered statistically significant. All analyses were conducted using Stata Version 14.1. 

Ethics approval: The survey protocols and all materials, including the survey questionnaires, were cleared for ethics by Institutional Review Board, Medical University of South Carolina (ORE #: 20803); Research Ethics Office, King’s College London, UK (ORE #: 20803 and RESCM-17/18-2240); Office of Research Ethics, University of Waterloo, Canada (ORE #: 20803 and ORE #: 21609); and Human Research Ethics, Cancer Council Victoria, Australia (ORE #: 21609 and HREC 1603).

## 3. Results

### 3.1. Sample Characteristics

Characteristics of the sample are provided in Table 1. Of the total analytic sample (*n* = 11,344), 3433 respondents were currently vaping (using at least monthly). There were more males than females in the sample. The Australian respondents were least likely to be current vapers, whereas respondents from England were most likely to be so. The majority of the sample were current smokers (smoked at least monthly). 

In multivariate analyses where all the measures in Table 1 were included together, we found that males were less likely to be current monthly vapers (adjusted OR, AOR = 0.84, 95% CI 0.73–0.96, *p* < 0.05; these results are not reported in the table), compared to females. Respondents from England were more likely to be monthly vapers (AOR = 1.52, 95% CI 1.32–1.76, *p* < 0.001), whereas Australian respondents were less likely to be so (AOR = 0.27, 95% CI 0.21–0.34, *p* < 0.001), compared to their Canadian/US counterparts. Current smokers were not more likely to be monthly vapers, compared to recent quitters (AOR = 0.96, 95% CI 0.80–1.14, *p* = 0.63). 

We repeated this analysis this time comparing daily vapers (*n* = 1425) with all others. We found that current smokers were less likely to be daily vapers (AOR = 0.46, 95% CI 0.37–0.57, *p* < 0.001), compared to quitters. Overall, those aged 40 and older were more likely to be daily vapers (AOR = 1.39, 95% CI 1.11–1.73, *p* < 0.01), compared to those under 40. Again, respondents from England were more likely to be daily vapers (AOR = 2.31, 95% CI 1.86–2.88, *p* < 0.001), whereas Australian respondents were less likely to be so (AOR = 0.39, 95% CI 0.28–0.54, *p* < 0.001). No gender differences were found in daily vaping (AOR = 0.90, 95% CI 0.74–1.10, *p* = 0.32).

### 3.2. Reporting of Health Conditions by Main Sample Characteristics

Table 2 presents percentages of health problems by sex and age. More people reported mental health conditions than physical conditions. Not surprisingly, the most severe physical conditions were generally reported at lower levels. The vast majority (87%) accepted that smoking had damaged their health, and 91% were concerned about future damage from smoking. 

There were some differences in rates of reporting health conditions/concerns about damage from smoking by the demographic variables. Women were more likely to report anxiety, depression, chronic pain, severe obesity, and chronic lung disease, while males were more likely to report alcohol problems, diabetes, heart disease, and more likely to accept that smoking had affected their health. Older people were more likely to report the physical health conditions but were less likely to report anxiety and depression. There were nonlinear relationships for severe obesity or alcohol problems. 

### 3.3. Current Vaping among Those with and without Health Problems

Of the total analytic sample, 3433 respondents were currently vaping at least monthly (which also included daily); and 1425 respondents were current daily vapers. Table 3 presents the overall results for both presence of current vaping (both daily and at least monthly) by condition, and intention (planning) to continue use among current monthly (at least) vapers. The percentages reported should be considered with care where there were significant interactions. For monthly vaping, the only significant interaction by condition was with age and cancer. By contrast, there were several interactions with daily vaping, as we describe below. 

Respondents with depression and alcohol problems were more likely to be current vapers both daily (AOR = 1.42, 95% CI 1.09–1.85 for depression, and AOR = 1.52, 95% CI 1.02–2.27 for alcohol) and monthly (AOR = 1.32, 95% CI 1.11–1.57 for depression, and AOR = 1.43, 95% CI 1.06–1.90 for alcohol). For severe obesity, vaping was more likely at the at least monthly level (AOR = 1.77, 95% CI 1.29–2.43), but this was not significant for daily where there were by condition interactions with smoking status (AOR = 2.55, 95% CI 1.04–6.26) and country (see Table 3 for details). When analyzed separately, we found that for current smokers, those with obesity (AOR = 2.80, 95% CI 1.99–3.94, *p* < 0.001) were more likely to be daily vapers compared to those without obesity; whereas for recent quitters, the differences between those with and without the condition were not significant (AOR = 1.32, 95% CI 0.58–3.03, *p* > 0.05); in Canada (AOR = 1.77, 95% CI 1.06–2.98, *p* < 0.05) and the US (AOR = 4.17, 95% CI 2.13–8.18, *p* < 0.001), those with severe obesity were more likely to be daily vapers, compared to those without the condition; but the differences were not significant in England (AOR = 1.43, 95% CI 0.63–3.19, *p* > 0.05) and Australia (AOR = 0.35, 95% CI 0.10–1.16, *p* > 0.05). By contrast, for chronic pain, vaping was more likely only at daily level (AOR = 2.95, 95% CI 1.86–4.67, *p* < 0.001), but there were by condition interactions with both sex (AOR = 0.48, 95% CI 0.27–0.78, *p* < 0.01) and country (Table 3). When analyzed separately, we found females with chronic pain (AOR = 1.52, 95% CI 1.07–2.18, *p* < 0.05) were more likely to be daily vapers compared to those without the condition; whereas for males, the differences between those with and without the condition were not significant (AOR = 0.90, 95% CI 0.59–1.37, *p* > 0.05).

Those with cancer (AOR = 5.19, 95% CI 2.20–12.24) were more likely to be current monthly vapers, although there was also an age by condition interaction. When analyzed separately, we found that younger cancer sufferers under 40 (AOR = 4.10, 95% CI 1.92–10.87, *p* < 0.01) were more likely to be monthly vapers compared to those without the condition; whereas for those aged 40 and over, the differences between those with and without the condition were not significant (AOR = 1.12, 95% CI 0.66–1.91, *p* > 0.05). For daily vaping, the relationship was not significant, although given the small number of cases with cancer, power is low. Those with concern about future health effects (smokers only) were more likely to be current monthly vapers (AOR = 1.83, 95% CI 1.47–2.28) but no more likely to be daily. The other significant positive effect was for those concerned that smoking had damaged their health where daily vaping was more likely (AOR = 2.63, 95% CI 1.47–4.68) in the context of significant by condition interactions with age (AOR = 0.56, 95% CI 0.32–0.99) and smoking status (AOR = 0.49, 95% CI 0.26–0.89, *p* < 0.05). When analyzed separately, we found those who were under 40 and with concern (AOR = 1.75, 95% CI 1.11– 2.73, *p*< 0.05) were more likely to be daily vapers compared to those without concern; and recent quitters with concern (AOR = 1.83, 95% CI 1.05–3.21, *p* < 0.05) were also more likely to be daily vapers compared to those without concern. Those with diabetes (AOR = 1.78, 95% CI 1.01–3.15) were marginally more likely to be daily vapers when controlling for a condition by age interaction. When analyzed separately, we found that for the group under 40, those with diabetes (AOR = 1.81, 95% CI 1.03–3.17, *p* < 0.05) were more likely to be daily vapers compared to those without the condition; whereas for the group aged 40 and over, those with diabetes (AOR = 0.53, 95% CI 0.33–0.83, *p* < 0.01) were less likely to be daily vapers.

In the supplementary analyses grouping conditions, those having “any” of the three mental health conditions (i.e., depression, anxiety and alcohol problem) were more likely to be current vapers, both daily (AOR = 1.35, 95% CI 1.06– 1.72, *p* < 0.05) and monthly (AOR = 1.40, 95% CI 1.20–1.60, *p* < 0.001), compared to those without. Having “any” of the three smoking-caused conditions (i.e., heart disease, cancer, and chronic lung disease) was not associated with either level of vaping. 

### 3.4. Planning to Keep Vaping among Current Vapers with and without Health Problems 

Overall, 58% of current monthly vapers (*n* = 3,433) said they planned to keep vaping. Current smokers were more likely to plan to keep vaping (AOR = 2.40, 95% CI 1.76–3.27, *p* < 0.001), compared to ex-smokers; and respondents from England (AOR = 1.72, 95% CI 1.33–2.23, *p* < 0.001) and the US (AOR = 1.43, 95% CI 1.04–1.97, *p* < 0.05) were more likely to plan than their counterparts in Canada and Australia; no sex differences were found in planning to keep vaping. The only significant effect found was an age by diabetes interaction effect (AOR = 0.28, 95% CI 0.13–0.60, *p* < 0.01, Table 3) (Note: When controlling for the condition by age interaction and covariates, overall, those with diabetes were not less likely to be planning to keep vaping). When analyzed separately, we found that among those 40 and over, those with diabetes (AOR = 0.50, 95% CI 0.30–0.79, *p* < 0.01) were less likely to plan to keep vaping than those without the condition; whereas for the group aged under 40, there was no significant difference (AOR = 1.59, 95% CI 0.89–2.86, *p* > 0.05). There was no significant association between “any mental health conditions” or “any smoking-caused conditions” and planning to keep vaping.

### 3.5. Use of Vaping Products at the Last Quit Attempt among Those with and without Health Problems

Finally, for those current daily smokers who reported having made at least one quit attempt within the last 12 months (*n* = 4339), we examined whether having a health condition was related to use of vaping products (see Table S1). The only association between individual conditions and use of vaping products on their last quit attempt was with heart disease, where use was lower for those with than those without the condition (17.42% vs. 27.71%, AOR = 0.72, 95% CI 0.43–0.91, *p* < 0.05).

## 4. Discussion

We did not observe strong consistent association between reported health problems and/or health concerns and vaping behaviors. However, we did find some potentially interesting associations with specific conditions and overall reporting of problems. In interpreting the results, some caution should be exercised, as there were large age-related effects on reporting most conditions, and strong age-related patterns of vaping, meaning in some cases very small absolute differences became significant when controlling for age and other sociodemographics, and/or quite large apparent differences became nonsignificant. We also note that the consistent finding that smokers were more likely to be vaping than the ex-smokers is, at least in part, due to sampling and dependence-related issues, and should not be over-interpreted. 

Because of the variability in the associations between vaping and the health conditions, we consider the findings by groupings of conditions. First, mental health related conditions. There was a clear positive association between reporting depression and alcohol problems and increased likelihood of current vaping, both daily and any current, but no clear trend for anxiety when treated separately. As depression and anxiety are related conditions, we might have expected a similar pattern. The differences may be because being anxiety inhibits trying vaping, perhaps because it is controversial, something anxiety-provoking in its own right, and/or because it is still a novel behavior and that also tends to evoke anxiety. This may mask a general increased interest in activities like vaping which may be thought to aid emotional regulation. It is also notable that in none of these conditions was vaping more likely to be used to quit.

In two conditions, severe obesity and chronic pain, smoking may be used in part of efforts to self-manage the problem. If so, vaping could be seen as an alternative strategy, one with fewer potential downsides. Overall, those with severe obesity were more likely to be currently vaping, but for daily use, this seems to be restricted to those from North America (Canada and the US), and not those from England and Australia. A similar condition by country interaction was found for daily vaping among those suffering from chronic pain. These were the only two condition by country interactions we found, hinting at something specific to conditions where smoking may play a significant self-management role (regardless of its efficacy). That said, it is unclear why this country difference occurs. Given that England and Australia are at opposite poles on the prevalence of vaping and its social acceptability, this difference is hard to interpret in terms of the different regulatory environment. There were also other different interactions for the two conditions. For obesity, the difference in daily use is almost entirely among current smokers, and for chronic pain, it was entirely among females. Again, in neither case were those with the condition more likely to use vaping products to quit.

Turning now to the three health conditions where smoking is a major cause (heart disease, chronic lung disease, and cancer), the only significant effect was for any current use for those with cancer, largely because young (under 40) cancer sufferers were much more likely to report some level of vaping, but there was no significant effect for daily use; however, the relatively large odds ratio suggests that this may be a function of low power rather than the necessary absence of any effect. It may be something about getting cancer at a young age that encourages a search for alternatives. There was a significant negative effect on use of vaping for heart disease. One possibility for the negative association with vaping as a cessation aid may be because of concerns about the role of use of nicotine as a cardiovascular stimulant and concerns about the risks of using it, particularly with people with unstable heart disease.

Turning now to diabetes, a group of diseases that is exacerbated by smoking. There was marginally more daily vaping in those with diabetes. However, there was a very strong age-related effect for both outcomes with those over 40 being both less likely to be daily vapers and less interested in continuing to vape, whereas for the under 40s, we found the reverse. We have no ready explanation for these findings but, given that diabetes is a chronic disease, it may be related to their long-term relationships with health professionals and/or knowledge that their smoking is likely to be increasing their risk of problems. However, this does not explain why those under 40 are more likely to be engaged with vaping while those over 40 are less likely.

Finally, those people who believe that smoking had already damaged their health were overall more likely to be daily vapers, but this only appeared to be the case for ex-smokers and for those under 40. For the related question (only asked to smokers) about concern that smoking may damage their health in future, the majority who expressed concern were more likely to be vaping at least monthly, but not daily, suggesting it may be motivating some experimental use of vaping products, but not high level use, and this is consistent with vaping not being more likely to be used to quit.

Given the differences above, it is unwise to draw any general conclusions. We think that the results point to the potential utility of exploring in more detail how the availability of vaping might be influencing smokers with mental health problems, people with diabetes, and more generally those who were concerned about the health damage their smoking has done or might be doing. 

The main weakness of this study is that it is reliant on self-report for the conditions and we have little information about the management of the diseases and the attitudes of their health professionals to vaping. Where issues identified here are of concern, or interest, they may need to be pursued with clinical samples. There were also differences in how NVP users were sampled in the continuing part of the sample compared to the replenished part, and although we tried to control for this with weighting, we cannot be sure the ex-smokers are fully representative as a function of vaping status. This precludes drawing any conclusions about differential effects on quit success. The main strength of the study is the broad focus across conditions and the comparisons we were able to make. There can be gaps between what respondents identify as having and what they have, which can be affected by the level of education about their conditions, the terminology used (and how it related to the terms used in our questions), and a range of other factors. These concerns are less of an issue in relating conditions that people self-report in relation to their smoking as where links exist, smokers with such conditions or at heightened risk of developing them should know about the additional risks they are subjecting themselves to.

## 5. Conclusions

In conclusion, having a health condition or reduced health associated with smoking is not systematically leading to increased vaping or increased likelihood of using vaping as a quitting strategy. Among those with some conditions, younger sufferers were more likely to be vaping, but not always regularly.

## Figures and Tables

**Table 1:** Sample characteristics by vaping status, % (95% confidence interval), weighted.

Vaping Status			
Characteristics	Not Currently Vaping	Currently Vaping (at least monthly)	Overall
	*n* = 7911	*n* = 3433	*n* = 11,344 ^
Sex **			
Female	43.3 (41.9, 44.8)	47.6 (44.7, 50.6)	43.8 (42.5, 45.2)
Male	56.7 (55.2, 58.2)	52.4 (49.4, 55.3)	56.2 (54.8, 57.5)
Age			
18–24	11.6 (10.6, 12.6)	12.9 (11.1, 15.1)	11.7 (10.9, 12.6)
25–39	34.4 (32.9, 35.9)	30.8 (28.1, 33.7)	34 (32.6, 35.4)
40–54	28.4 (27.1, 29.7)	30.8 (28.2, 33.6)	28.7 (27.5, 29.9)
≥55	25.7 (24.6, 26.8)	25.4 (23.1, 27.9)	25.6 (24.6, 26.7)
Education			
Low	30.2 (28.9,31.6)	27.8 (25.4, 30.3)	29.9 (28.7,31.1)
Moderate	49.5 (48.1, 50.9)	52.2 (49.3, 55.1)	49.8 (48.5, 51.1)
High	20.3 (19.2,21.4)	19.9 (18.1, 22.1)	20.3 (19.3, 21.2)
Income			
Low	22.8 (21.6, 24.1)	22.7 (20.5, 25.1)	22.8 (21.7,24.0)
Moderate	27.8 (26.6, 29.2)	30.4 (27.8, 33.2)	28.1 (27.0, 29.3)
High	42.5 (40.9, 43.9)	41.1 (38.1, 44.1)	42.2 (40.9, 43.6)
No information	6.8 (6.1, 7.6)	5.8 (4.5, 7.5)	6.72 (6.07, 7.43)
Country ***			
Canada	30.5 (29.5, 31.5)	28.9 (26.8, 30.9)	30.2 (29.4, 31.1)
US	20.3 (19.3, 21.3)	17.5 (15.6, 19.5)	19.9 (19.1, 20.7)
England	34.6 (33.4, 35.7)	49.9 (47.1, 52.8)	36.5 (35.4, 37.4)
Australia	14.7 (13.8, 15.6)	3.7 (3.1, 4.5)	13.2 (12.5, 14.0)
Cigarette smoking status			
Recent quitters	32.5 (30.8, 34.1)	34.4 (30.9, 37.9)	32.7 (31.2, 34.2)
Current smokers (at least monthly)	67.5 (65.9, 69.1)	65.7 (62.1, 69.1)	67.3 (65.8, 68.8)

^ In some analyses, the sample size was smaller than the total due to missing cases. ** significant at *p* < 0.01; *** *p* < 0.001.

**Table 2:** Percentages reporting health conditions, by sex and age (total *n* = 11,344 ^), weighted.

Health Condition	Sex		Age				Total
	Female (*n* = 5465)	Male (*n* = 5879)	18–24 (*n* = 1840)	25–39 (*n* = 2988)	40–54 (*n* = 3097)	55+ (*n* = 3419)	*n* = 11.344
**Depression** (% yes)	24.9	16.8	24.0	22.1	22.7	13.9	20.4
OR (95% CI)#	Ref	0.6 (0.6–0.7) ***	Ref	1.1 (0.8–1.4)	1.1 (0.8–1.4)	0.5 (0.4–0.7) ***	
**Anxiety** (% yes)	25.4	14.2	26.1	22.4	18.6	12.1	19.1
AOR (95% CI)	Ref	0.5 (0.4–0.6) ***	Ref	0.9 (0.7–1.1)	0.7 (0.5–0.9)**	0.4 (0.3–0.5) ***	
**Alcohol problem** (% yes)	1.4	3.2	2.4	2.4	2.7	2.0	2.4
AOR (95% CI)	Ref	2.4 (1.7–3.3) ***	Ref	1.1 (0.7–1.7)	1.1 (0.7–1.7)	0.7 (0.5–1.2)	
**Severe obesity** (% yes)	4.2	2.6	2.9	3.2	4.8	2.1	3.3
AOR (95% CI)	Ref	0.6 (0.5–0.9) **	Ref	1.1 (0.7–1.9)	1.7 (1.1–2.9) *	0.7 (0.4–1.1)	
**Chronic pain** (% yes)	12.5	9.9	4.5	7.3	14.5	15.4	11.1
AOR (95% CI)	Ref	0.8 (0.7–0.9) **	Ref	1.8 (1.1–3.1) *	3.8 (2.3–6.3) ***	3.7 (2.2–6.1) ***	
**Diabetes** (% yes)	5.9	9.3	2.2	3.2	9.0	15.1	7.8
AOR (95% CI)	Ref	1.6 (1.3–1.9) ***	Ref	1.3 (0.6–2.8)	4.1 (2.1–8.3) ***	7.1 (3.5–14.1) ***	
**Heart disease** (% yes)	2.1	5.5	0.9	1.1	3.5	9.9	4.1
AOR (95% CI)	Ref	2.8 (2.1–3.7) ***	Ref	0.9 (0.4–2.2)	3.2 (1.7–6.4) **	9.3 (4.9–17.5) ***	
**Cancer** (%yes)	1.9	2.0	0.8	0.4	1.9	4.6	1.9
AOR (95% CI)	Ref	1.0 (0.7–1.4)	Ref	0.5 (0.2–1.2)	2.4 (1.1–5.4) *	5.8 (2.7–12.2) ***	
**Lung disease** (%yes)	7.2	5.6	5.1	3.4	6.3	10.7	6.3
AOR (95% CI)	Ref	0.8 (0.6–0.9) *	Ref	0.7 (0.5–1.2)	1.4 (0.9–2.1)	2.2 (1.5–3.2) ***	
**Smoking has damaged health** (% yes)	85.1	88.6	79.4	87.7	89.3	87.3	87.1
AOR (95% CI)	Ref	1.4 (1.2–1.6) ***	Ref	1.8 (1.4–2.4) ***	2.1 (1.6–2.7) ***	1.7 (1.3–2.2) ***	
**Smoking will damage health** (% yes)	91.2	90.9	87.7	92.5	91.9	89.8	91.1
AOR (95% CI)	Ref	0.9 (0.8–1.1)	Ref	1.7 (1.2–2.3) **	1.5 (1.1–2.1) **	1.2 (0.9–1.6)	

^ In some analyses, the sample size was smaller than the total due to missing cases. #Logistic regression results, with female/18–24 years old as the reference value (ref), controlling for age (sex), education, income, and other covariates as indicated in methods. AOR: Adjusted odds ratio; CI: Confidence interval. * Significant at *p* < 0.05; ** *p* < 0.01; *** *p* < 0.001.

**Table 3:** Association between self-reported health problems and vaping activities (in all 4 countries) (weighted).

Health Condition	~% Currently Daily Vaping (*n* = 11,344 ^)	~% Currently Monthly Vaping (*n* = 11,344 ^)	~% Planning to Keep Vaping among Current Monthly Vapers (*n* = 3433 ^)
Overall	6.2	12.5	58.4
Depression			
No (Ref)	5.8	11.9	59.3
Yes	7.8	15.1	55.4
Adjusted OR (95% CI) #	1.42 (1.09–1.85) *	1.32 (1.11–1.57) **	0.87 (0.64–1.19)
Anxiety			
No (Ref)	6.2	12.3	57.5
Yes	5.9	13.7	61.8
Adjusted OR (95% CI)	0.96 (0.73–1.26)	1.16 (0.97–1.38)	1.27 (0.93–1.71)
Alcohol problem			
No (Ref)	6.1	12.4	58.1
Yes	7.2	15.1	66.8
Adjusted OR (95% CI)	1.52 (1.02–2.27) *	1.43 (1.06–1.90) *	1.29 (0.74–2.25)
Severe obesity			
No (Ref)	6.0	12.3	58.6
Yes	10.6	18.6	54.5
Adjusted OR (95% CI) (main effect)	1.08 (0.55–2.14)	1.77 (1.29–2.43) ***	0.90 (0.54–1.50)
Interactions with condition: Country (CA as ref) ##	US = 2.09 (0.89–5.10); EN = 0.74 (0.29–1.89);		
	AU = 0.19 (0.06–0.64) **		
Smoking status (quit as ref ) ##	2.55 (1.04–6.26) *		
Chronic pain			
No (Ref)	6.1	12.3	58.1
Yes	7.1	14.2	60.3
Adjusted OR (95% CI) (main effect)	2.95 (1.86–4.67) ***	1.23 (1.00–1.49)	1.10 (0.78–1.54)
Interactions with condition: Sex (female as ref) ##	0.48 (0.27–0.78) **	N/A	N/A
Country (CA as ref)	US = 0.66 (0.34–1.30); EN = 0.37 (0.20–0.70) **; AU = 0.29 (0.12–0.66) **		
Diabetes			
No (Ref)	6.3	12.7	58.8
Yes	4.5	10.9	53.3
Adjusted OR (95% CI) (main effect)	1.78 (1.01–3.15) *	0.82 (0.64–1.04)	1.75 (0.98–3.13)
Interactions with condition: Age (< 40 as ref) ##	0.27 (0.13–0.56) ***	N/A	0.28 (0.13–0.60) **
Heart disease			
No (Ref)	6.1	12.6	58.4
Yes	7.3	12.3	57.4
Adjusted OR (95% CI)	1.15 (0.76–1.74)	1.03 (0.74–1.43)	0.97 (0.55–1.73)
Cancer			
No (Ref)	6.1	12.5	58.2
Yes	10.8	17.9	54.4
Adjusted OR (95% CI) (main effect)	1.57 (0.85–2.91)	5.19 (2.20–12.24) ***	0.92 (0.41–2.05)
Interactions with condition: Age (< 40 as ref)	N/A	0.23 (0.08–0.62) **	N/A
Chronic lung disease			
No (Ref)	6.1	12.5	58.4
Yes	7.7	14.3	54.2
Adjusted OR (95% CI)	0.96 (0.67–1.39)	1.1 1(0.87–1.43)	0.74 (0.47–1.17)
Smoking has damaged health			
No (Ref)	5.1	11.1	56.3
Yes	6.3	12.8	58.5
Adjusted OR (95% CI) (main effect)	2.63 (1.47–4.68) **	1.17 (0.95–1.43)	1.09 (0.77–1.56)
Interactions with condition: Smoking status (quit as ref)	0.49 (0.26–0.89) *		
Age (<40 as ref)	0.56 (0.32–0.99) *	N/A	N/A
Smoking will damage health			
No (Ref)	3.7	7.4	59.9
Yes	4.7	12.4	62.9
Adjusted OR(95% CI)	1.31 (0.96–1.79)	1.83 (1.47–2.28) ***	1.09 (0.72–1.63)

^ In some analyses, the sample size was smaller than the total due to missing cases. # Logistic regression results; all odds ratios (ORs) were adjusted for sex, age, education, income, smoking status, and country; “no” health problem group as reference value (ref); CI: confidence interval. This applies to all other health problems; ## This is by health condition interaction with country/smoking status/sex/age. * Significant at *p* < 0.05; ** *p* < 0.01; *** *p* < 0.001. N/A: Not asked/applicable.

## Supplementary Materials

The following are available online at https://www.mdpi.com/1660-4601/16/8/1412/s1, Table S1: Association between self-reported health problems and use of vaping on the last quit attempt among current daily smokers.
