# Supplementary material for: How Are Self-Reported Physical and Mental Health Conditions Related to Vaping Activities among Smokers and Quitters: Findings from the ITC Four Country Smoking and Vaping Wave 1 Survey
Source: Int J Environ Res Public Health. 2019 Apr 19;16(8):1412. doi: 10.3390/ijerph16081412 (PMC6518008; doi:10.3390/ijerph16081412)
Supplement: Supplementary file 1 [file ijerph-16-01412-s001.pdf]

Table S1. Association between self-reported health problems and use of vaping on the last quit attempt among current daily smokers (n = 4399, in all 4 countries).

Health condition	Use of vaping products to quit (% yes)
Overall	27.23
**Depression**	(Ref)
No	26.41
Yes	30.43
Adjusted OR(95%CI) #	1.12 (0.91–1.43)
**Anxiety**	(Ref)
No	26.83
Yes	29.42
Adjusted OR(95%CI)	1.12 (0.81–1.34)
**Alcohol problem**	(Ref)
No	27.41
Yes	22.12
Adjusted OR(95%CI)	0.81 (0.51–1.22)
**Severe obesity**	(Ref)
No	27.32
Yes	28.71
Adjusted OR(95%CI)	0.91 (0.62–1.53)
**Chronic pain**	(Ref)
No	27.62
Yes	25.13
Adjusted OR(95%CI)	0.93 (0.72–1.23)
**Diabetes**	(Ref)
No	27.83
Yes	21.34
Adjusted OR(95%CI)	0.82 (0.62–1.13)
**Heart disease**	(Ref)
No	27.71
Yes	17.42
Adjusted OR(95%CI)	0.72 (0.43–0.91)*
**Cancer**	(Ref)
No	27
Yes	33.12
Adjusted OR(95%CI)	1. 51 (0.82–2.81)
**Chronic lung disease**	(Ref)
No	27.12
Yes	28.13
Adjusted OR(95%CI)	1.00 (0.71–1.52)
**Smoking has damaged health**	(Ref)
No	32.31
Yes	26.73
Adjusted OR(95%CI)	0.82 (0.63–1.01)
**Smoking will damage health**	(Ref)
No	22.42
Yes	28.41
Adjusted OR(95%CI)	1.32 (0.83–2.14)

^ In some analyses, the sample size was smaller than the total due to missing cases. #Logistic regression results; all odds ratios (ORs) were adjusted for sex, age, education, and income; "no" health problem group as reference value (ref). This applies to all other health problems. *Significant at $p < 0.05$; **$p < 0.01$; *** $p < 0.001$.
